# Impact of closed-system suctioning on self-reported dyspnea in mechanically ventilated patients: a prospective observational study

**DOI:** 10.3389/fmed.2026.1834389

**Published:** 2026-06-23

**Authors:** Lijun Liang, Simei Wang, Zhenghua Liang, Xiaoli Qiu, Sha Xie, Jinlong Xu

**Affiliations:** Intensive Care Unit, Mianyang Central Hospital, School of Medicine, University of Electronic Science and Technology of China, Mianyang, Sichuan, China

**Keywords:** critical care nursing, dyspnea, endotracheal suctioning, intensive care, mechanical ventilation, patient-reported outcomes, symptom assessment

## Abstract

**Background:**

Endotracheal suctioning is an essential airway care procedure in mechanically ventilated patients, yet its acute impact on subjective respiratory distress has not been systematically quantified.

**Methods:**

In this prospective self-controlled observational study conducted from May to October 2025 in a tertiary intensive care unit (ICU) in Sichuan Province, China, awake mechanically ventilated adults with a Richmond Agitation-Sedation Scale (RASS) score of 0 to +1 requiring closed-system suctioning were enrolled. Dyspnea intensity was assessed using the Dyspnea Visual Analog Scale (D-VAS, 0–10) before suctioning (T0), immediately after suctioning (T1), and 5 min post-suctioning (T2). Peripheral oxygen saturation (SpO_2_) and heart rate (HR) were recorded simultaneously.

**Results:**

Fifty-three suctioning procedures from 42 patients were included. D-VAS scores increased significantly from 2.8 ± 1.9 at T0 to 6.4 ± 2.1 at T1 (mean increase 3.6, 95% CI: 3.1–4.1, *P* < 0.001), then decreased to 3.2 ± 1.7 at T2 (*P* < 0.001 vs. T1). Dyspnea worsened in 92.5% of procedures at T1, and 15.1% had not returned to baseline at T2. Despite SpO_2_ increasing from 97.2 ± 2.1% to 99.1 ± 1.3% at T1, dyspnea worsened substantially, suggesting a potential dissociation between oxygenation status and subjective respiratory distress. These findings should be interpreted within the context of a single-center convenience sample without prospective sample size calculation and the use of a unidimensional dyspnea instrument.

**Conclusion:**

These findings suggest that closed-system suctioning may induce clinically significant but transient worsening of dyspnea that appears independent of oxygenation status. Individual dyspnea assessment during routine suctioning care warrants attention, and confirmatory multicenter studies with prospective power calculations are needed.

## 1 Introduction

Dyspnea is reported by 33%–95% of mechanically ventilated patients and is frequently described as one of the most traumatic experiences during ICU stay (1–3). Schmidt et al. (4) found that 47% of communicative mechanically ventilated patients reported dyspnea, with a median intensity of 5 on a visual analog scale (VAS, 0–10), and dyspnea was significantly associated with anxiety (odds ratio 8.84). As an essential nursing procedure for maintaining airway patency, endotracheal suctioning may directly trigger or exacerbate respiratory distress due to its invasive nature and dramatic disturbance to airway physiology. Despite its clinical ubiquity, the acute impact of suctioning on patients' subjective respiratory experience has not been systematically quantified.

The mechanisms by which suctioning may induce dyspnea are hypothesized to be multidimensional. Neuro-mechanical uncoupling—a severe mismatch between efferent commands from the respiratory center and afferent feedback from the respiratory system—has been proposed as one core mechanism (5). Additional proposed mechanisms—including chemoreceptor activation following alveolar derecruitment, afferent airway receptor stimulation, and downstream affective amplification—are discussed in the context of our observed findings in the Discussion. Although these mechanisms are supported by neurophysiological evidence, they remain speculative in the specific context of suctioning without direct physiological monitoring.

Although pain management has established relatively mature assessment and intervention systems in the ICU, dyspnea remains less consistently recognized or addressed, and physiological monitoring indicators often fail to reflect patients' internal respiratory experience (6). We therefore quantified changes in self-reported dyspnea during closed-system suctioning in awake mechanically ventilated patients and examined how these changes related to simultaneous physiological parameter changes.

## 2 Materials and methods

### 2.1 Study design and ethical considerations

This was a prospective self-controlled observational study using a within-subject repeated measures design. The study was conducted from May to October 2025 in the general ICU of a tertiary hospital in Sichuan Province, China. This study was approved by the Biomedical Ethics Committee of Mianyang Central Hospital (Approval No. S20250249-01) and conducted in accordance with the Declaration of Helsinki. Given that this was a non-interventional observational study with no deviation from standard care, the ethics committee specifically approved the use of verbal informed consent. Verbal consent was obtained by the research team member from each patient (or their legally authorized representative when patients had reduced communication capacity) before enrollment, and was documented in the patient's medical record along with the date, time, and witnessing nurse signature.

### 2.2 Participants

Inclusion criteria were: age ≥18 years, established artificial airway with ongoing mechanical ventilation, awake and communicative (RASS score 0 to +1), clinical indication for suctioning (rhonchi, ventilator high-pressure alarm, or SpO_2_ decline), and ability to express dyspnea severity through gestures. Patients receiving continuous sedative infusions (e.g., propofol, dexmedetomidine, midazolam) were eligible for inclusion provided their RASS remained 0 to +1 at the time of assessment, ensuring adequate awareness for self-report. Detailed records of the specific timing, dosing, and cumulative exposure of sedative agents prior to enrollment were not systematically extracted, a limitation acknowledged below.

Exclusion criteria included: drowsiness or deep sedation (RASS ≤ –1), severe agitation (RASS ≥+2), hemodynamic instability, emergency resuscitative suctioning, and known cognitive impairment (e.g., dementia, Alzheimer's disease, or any other condition precluding reliable communication or understanding of the D-VAS rating task). The same patient could contribute data from multiple suctioning procedures, provided procedures were separated by at least 24 h. The 24-h minimum inter-procedure interval was selected based on three considerations: (a) typical clinical suctioning frequency in stable mechanically ventilated patients in our ICU, which routinely allows extended intervals between non-urgent suctioning events; (b) the need to allow physiological and psychological recovery from any procedure-related distress; and (c) practical avoidance of anticipatory anxiety carry-over between closely spaced assessments. Within-patient correlation arising from repeated measurements was formally addressed using linear mixed-effects models, as described below. No formal *a priori* sample size calculation was performed; this was a convenience sample enrolling all eligible procedures during the 6-month study period.

### 2.3 Suctioning procedures

All suctioning procedures were performed according to institutional standard protocols, referencing the American Association for Respiratory Care (AARC) clinical practice guidelines (7), using a closed-system suctioning device (14Fr). Procedural steps included: (a) pre-oxygenation with FiO_2_ 1.0 for 2 min prior to catheter insertion. We note that this duration is longer than the 30–60 s suggested by AARC guidelines; the 2-min protocol reflects long-standing institutional practice intended to ensure adequate oxygen reserve in our heterogeneous ICU patient population, particularly those with borderline oxygenation. The implications of this extended pre-oxygenation for SpO_2_ interpretation are addressed in the Discussion and Limitations sections; (b) insertion of the suction catheter to the carina or until resistance was met; (c) application of continuous negative pressure (80–120 mmHg) during withdrawal, with single suction duration ≤ 15 s. Each suctioning procedure consisted of one to three catheter insertions as clinically indicated by secretion clearance and operator judgment; the mean reported procedure duration of 42.3 ± 18.6 s reflects the cumulative time across all insertions within a single procedure. Saline lavage was not routinely performed during the study procedures.

### 2.4 Outcome measures and data collection

The primary outcome was dyspnea intensity, measured using the D-VAS (0–10). The D-VAS has been validated in mechanically ventilated ICU patients (8), and an increase of 1–2 points is considered the minimal clinically important difference (MCID) (9). Secondary outcomes included SpO_2_ and HR.

Measurements were performed at three predefined time points: T0 (baseline before suctioning), T1 (as soon as the initial cough reflex had subsided, within a maximum of 30 s after catheter withdrawal), and T2 (5 min post-suctioning). T0 measurements (D-VAS, SpO_2_, and HR) were recorded immediately before the initiation of pre-oxygenation, representing the patient's baseline status under routine ventilatory settings without supplemental oxygen elevation. T1 SpO_2_ values therefore reflect the combined effects of pre-oxygenation and the suctioning procedure, rather than the suctioning procedure alone. Bedside nurses assessed dyspnea using a standardized prompt: “Are you having difficulty breathing right now? 0 means not difficult at all, 10 means the most difficult.” Patients indicated their level by pointing to the corresponding number or nodding. Patient demographics, primary diagnosis, artificial airway type, duration of mechanical ventilation, ventilator parameters, and suctioning duration were also collected.

In our staffing model, D-VAS assessment was performed by the bedside clinical nurse who also performed the suctioning procedure; deployment of an independent observer for every procedure was not logistically feasible given ICU workflow constraints. To minimize potential reporter bias arising from this design, several mitigation measures were implemented: (a) a standardized verbal prompt was used for all assessments ("Are you having difficulty breathing right now? 0 means not difficult at all, 10 means the most difficult."), with no leading or interpretive elaboration permitted; (b) patients indicated their score by pointing to a printed 0–10 numerical scale held at the bedside, rather than by verbal response or gesture to the operator, reducing social-desirability pressure; (c) all participating nurses completed pre-study training on neutral prompt delivery, and a written standard operating procedure was posted at each bedside; (d) the same nurse performed all three measurements (T0, T1, and T2) for a given procedure to ensure within-procedure consistency. Patient demographics and ventilator parameters were extracted from the electronic medical record after each procedure. We explicitly acknowledge in the Limitations section that complete blinding of the assessor from the procedure performer was not achieved and discuss the implications for interpretation.

Approximately 30 s before each procedure, the bedside nurse used a standardized statement to inform the patient: “We are about to perform airway suctioning. I will ask you to rate your breathing difficulty before, immediately after, and five minutes after the procedure, using a 0–10 scale by pointing or nodding." This standardized pre-procedure communication ensured uniform expectations across all enrolled procedures and minimized variability arising from different communication styles among nurses.

### 2.5 Statistical analysis

Statistical analysis was performed using R version 4.4.2 (R Foundation for Statistical Computing, Vienna, Austria). Continuous variables were expressed as mean ± standard deviation (SD) or median [interquartile range (IQR)] depending on distribution; categorical variables were reported as frequencies and percentages. The primary analysis used repeated measures analysis of variance (RM-ANOVA) to compare D-VAS scores across the three time points, with Bonferroni correction for *post-hoc* pairwise comparisons. Mauchly's test was used to assess sphericity; Greenhouse-Geisser correction was applied when violated. Secondary analyses of SpO_2_ and HR used the same methods.

Pearson or Spearman correlation analysis was used to explore relationships between dyspnea changes and physiological parameters. Linear mixed-effects models were used for sensitivity analysis to control for clustering effects of multiple observations from the same patient. Partial eta-squared ($\eta_{p}^{2}$) was calculated as the effect size measure (small: $\eta_{p}^{2}$ = 0.01; medium: $\eta_{p}^{2}$ = 0.06; large: $\eta_{p}^{2}$ = 0.14). Two-sided *P* < 0.05 was considered statistically significant. The large observed effect size for the primary outcome ($\eta_{p}^{2}$ = 0.71) suggests adequate statistical power to detect the primary endpoint despite the absence of an a priori sample size calculation.

## 3 Results

### 3.1 Patient characteristics

During the 6-month study period, 61 closed-system suctioning procedures from 48 eligible patients were screened. Eight procedures were excluded (five emergency resuscitative suctioning, two patient refusal of D-VAS assessment, one incomplete data recording), leaving 53 procedures from 42 patients in the final analysis. Among the 42 included patients, most contributed a single procedure, while the remaining patients contributed two or three procedures over the 6-month period (maximum three procedures per patient). Mean patient age was 58.6 ± 14.2 years; 26 (61.9%) were male; and mean BMI was 23.4 ± 3.8 kg/m^2^. Primary diagnoses included respiratory failure in 17 (40.5%), post-operative monitoring in 9 (21.4%), sepsis in 7 (16.7%), and neurological diseases in 6 (14.3%). The predominant artificial airway type was oral endotracheal intubation (83.3%). Median duration of mechanical ventilation at enrollment was 7 days (IQR: 4–12 days). Ventilation modes were mainly assist-control ventilation (A/C; 47.6%) and pressure support ventilation (PSV; 45.2%), with mean positive end-expiratory pressure (PEEP) of 6.2 ± 2.1 cmH_2_O and median FiO_2_ of 40% (IQR: 35%–50%). Mean suctioning duration was 42.3 ± 18.6 s, with 73.6% of procedures lasting < 60 s (Table 1).

**Table 1:** Baseline characteristics of study subjects (*n* = 42).

Characteristic	Value
Age (years)	58.6 ± 14.2
Male, *n* (%)	26 (61.9)
BMI (kg/m^2^)	23.4 ± 3.8
RASS score	0 (0 to +1)
APACHE II score	16.8 ± 5.2
Duration of mechanical ventilation (days)	7 (4–12)
Primary diagnosis, *n* (%)	
Respiratory failure	17 (40.5)
Post-operative monitoring	9 (21.4)
Sepsis	7 (16.7)
Neurological diseases	6 (14.3)
Other	3 (7.1)
Artificial airway type, *n* (%)	
Oral endotracheal intubation	35 (83.3)
Tracheostomy	7 (16.7)
Ventilation mode, *n* (%)	
A/C	20 (47.6)
PSV	19 (45.2)
Other	3 (7.1)
PEEP (cmH_2_*O*)	6.2 ± 2.1
FiO_2_ (%)	40 (35–50)
Suctioning duration (seconds)	42.3 ± 18.6

Continuous variables expressed as mean ± SD or median (IQR); categorical variables expressed as frequency (percentage).

BMI, body mass index; PEEP, positive end-expiratory pressure; A/C, assist-control ventilation; PSV, pressure support ventilation.

### 3.2 Changes in dyspnea intensity

**Figure 1:**
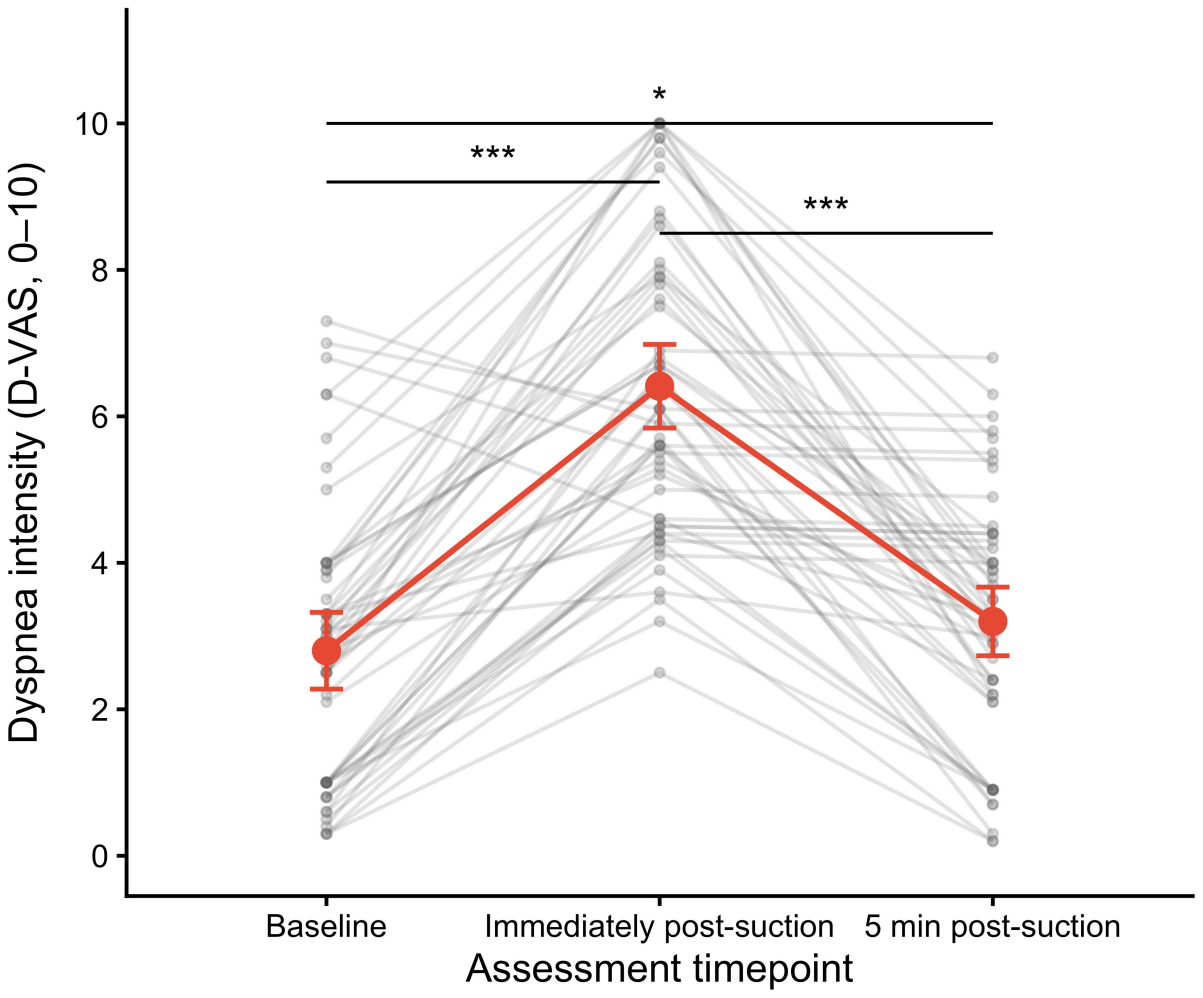
Dyspnea score changes across three time points. Gray points and connecting lines show individual trajectory changes for each of the 53 procedures before suctioning (T0), immediately after suctioning (T1), and 5 min post-suctioning (T2). Red points and lines show mean D-VAS scores. Error bars represent standard deviations. Exact *P*-values from Bonferroni-corrected pairwise comparisons: T0 vs. T1, *P* < 0.001; T1 vs. T2, *P* < 0.001; T0 vs. T2, *P* = 0.048. ^*^P < 0.05; ^***^P < 0.001.

Mauchly's test indicated violation of the sphericity assumption (*W* = 0.682, *P* = 0.001), so Greenhouse-Geisser correction was applied. Dyspnea scores differed significantly across the three time points (*F* = 127.84, *P* < 0.001, $\eta_{p}^{2}=0.71$, large effect). Mean D-VAS increased significantly from 2.8 ± 1.9 at T0 to 6.4 ± 2.1 at T1 (mean difference 3.6, 95% CI: 3.1–4.1, *P* < 0.001), then decreased to 3.2 ± 1.7 at T2, significantly lower than T1 (mean difference 3.2, 95% CI: 2.7–3.7, *P* < 0.001), but remaining slightly higher than T0 in some patients (mean difference 0.4, 95% CI: 0.03–0.77, *P* = 0.048; Figure 1).

At baseline, 18 (34.0%) procedures had D-VAS scores ≤ 1, 27 (50.9%) had scores of 2–4, and 8 (15.1%) had scores ≥5. Dyspnea scores increased in 49 (92.5%) procedures from T0 to T1, and scores decreased in all 53 (100%) procedures from T1 to T2. Net change from T0 to T2 showed: 31 (58.5%) procedures improved below baseline, 14 (26.4%) returned to baseline, and 8 (15.1%) remained above baseline.

### 3.3 Changes in physiological parameters

**Figure 2:**
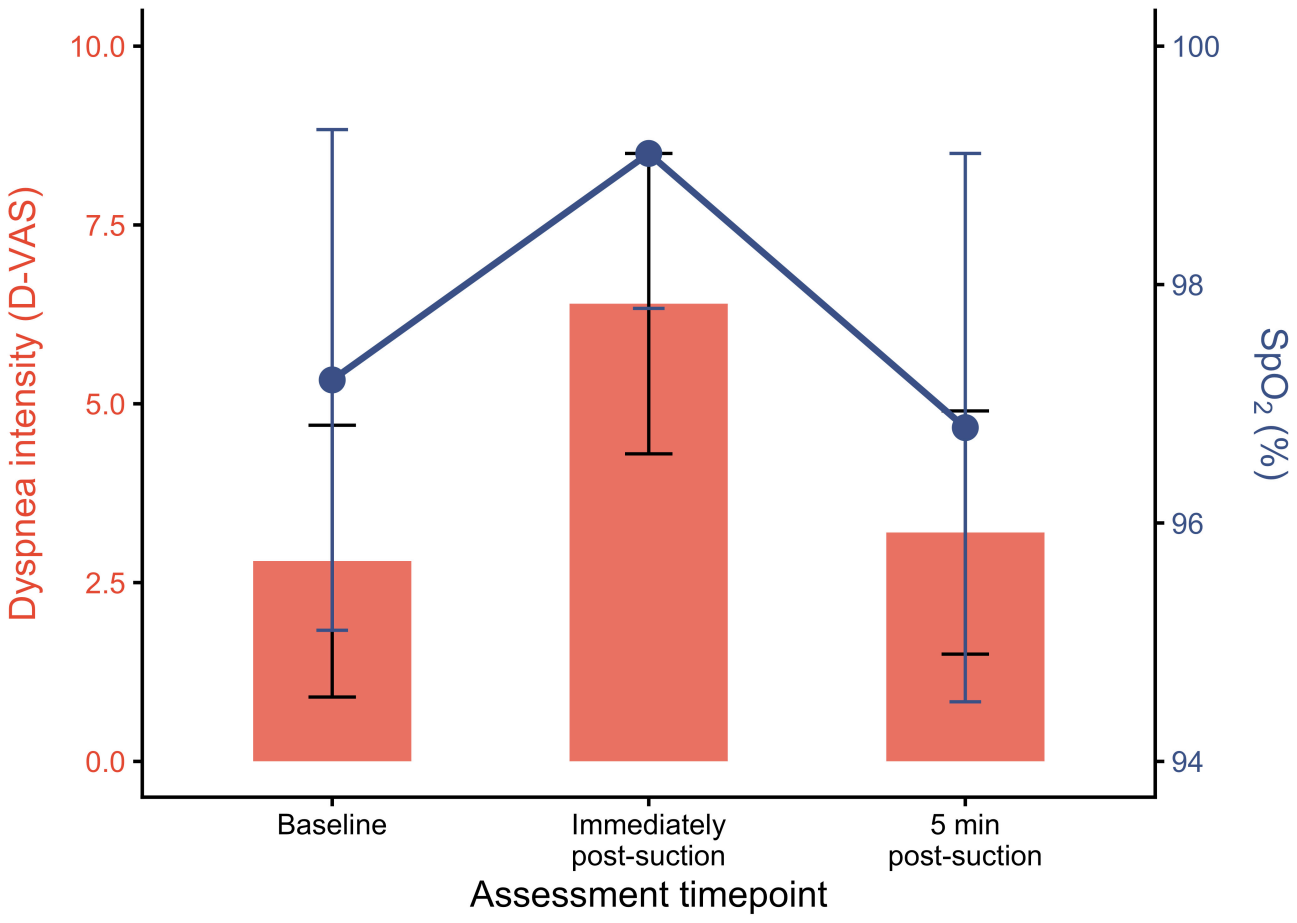
Dissociation between dyspnea and oxygenation. Dual Y-axis plot showing D-VAS scores (left axis, red) and SpO_2_ (right axis, blue) across T0, T1, and T2. At T1, SpO_2_ peaked (99.1%) while dyspnea scores also reached their maximum (6.4), visually demonstrating the dissociation between the two parameters. Error bars represent standard deviations.

SpO_2_ differed significantly across time points (*F* = 52.18, *P* < 0.001, $\eta_{p}^{2}=0.50$). SpO_2_ increased from 97.2 ± 2.1% at T0 to 99.1 ± 1.3% at T1 (mean difference +1.9%, 95% CI: +1.4 to +2.4, *P* < 0.001), then decreased to 96.8 ± 2.3% at T2 (*P* = 0.031 vs. T0). At T1, SpO_2_ reached ≥98% in 44 (83.0%) procedures, with 28 (52.8%) reaching 100%. HR increased from 79.3 ± 14.6 beats/min at T0 to 94.8 ± 16.2 beats/min at T1 (mean difference +15.5 beats/min, 95% CI: +12.5 to +18.5, *P* < 0.001), then recovered to 81.7 ± 15.1 beats/min at T2 (Table 2, Figure 2).

**Table 2:** Physiological parameter changes across time points.

Parameter	T0	T1	T2	*F*	*P*	$\eta_{p}^{2}$
D-VAS	2.8 ± 1.9	6.4 ± 2.1	3.2 ± 1.7	127.84	< 0.001	0.71
SpO_2_ (%)	97.2 ± 2.1	99.1 ± 1.3	96.8 ± 2.3	52.18	< 0.001	0.50
HR (beats/min)	79.3 ± 14.6	94.8 ± 16.2	81.7 ± 15.1	35.72	< 0.001	0.41

Data expressed as mean ± SD. *P* values from repeated measures ANOVA with Greenhouse–Geisser correction where indicated.

D-VAS, Dyspnea Visual Analog Scale; SpO_2_, peripheral oxygen saturation; HR, heart rate; $\eta_{p}^{2}$, partial eta-squared. Exact *P*-values from Bonferroni-corrected pairwise comparisons: D-VAS: T0 vs. T1, *P* < 0.001; T1 vs. T2, *P* < 0.001; T0 vs. T2, *P* = 0.048. SpO_2_: T0 vs. T1, *P* < 0.001; T0 vs. T2, *P* = 0.031. HR: T0 vs. T1, *P* < 0.001; T1 vs. T2, *P* < 0.001.

### 3.4 Subgroup analysis and correlations

**Figure 3:**
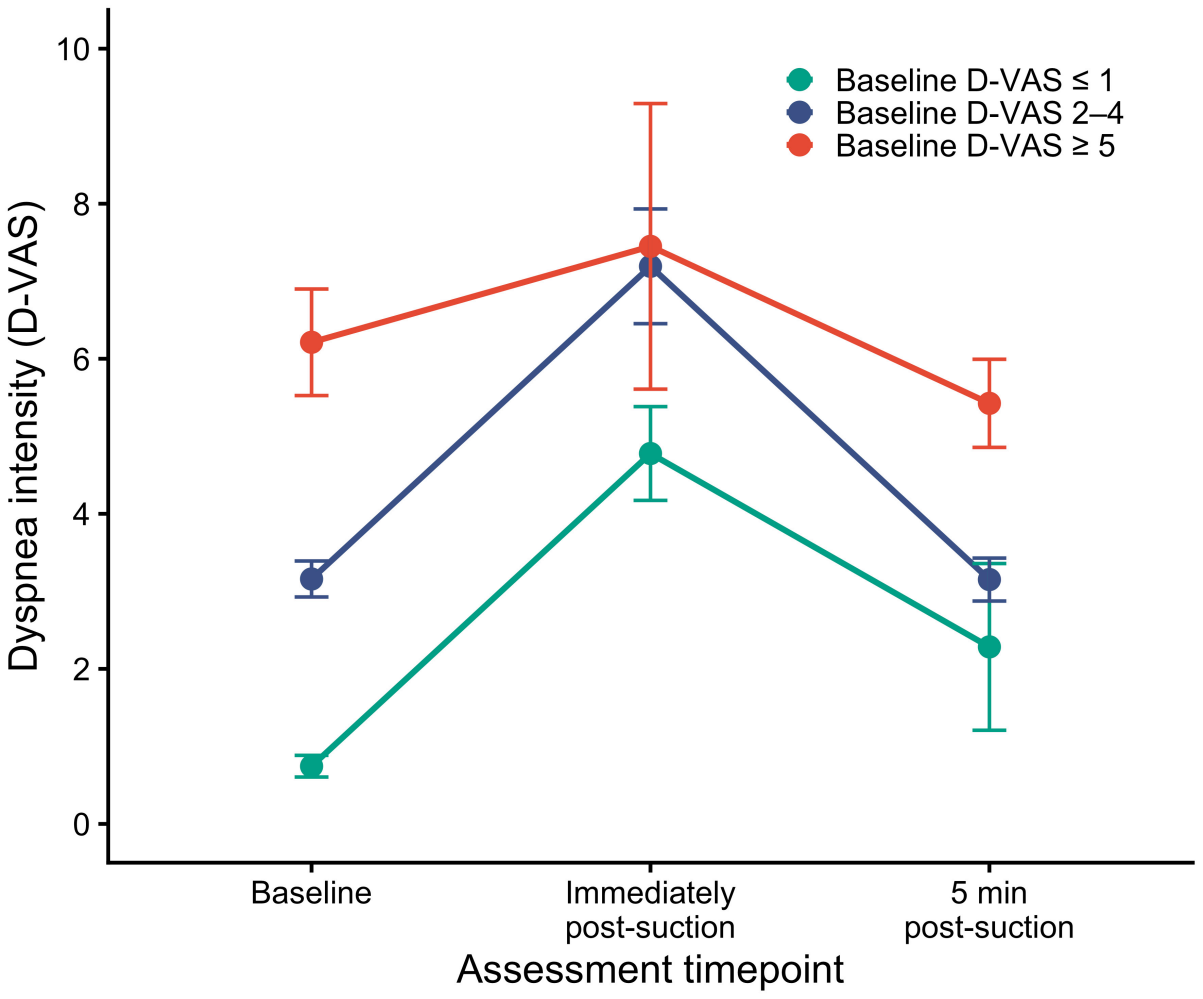
Recovery trajectories by baseline dyspnea stratification. Dyspnea change trajectories across T0, T1, and T2 stratified by baseline D-VAS score ( ≤ 1, 2–4, ≥5). The low baseline group ( ≤ 1) showed the largest increase at T1 but the most complete recovery; the high baseline group (≥5) showed limited increase due to a ceiling effect and slower recovery. Error bars represent standard deviations.

Stratification by baseline dyspnea severity showed that the D-VAS ≤ 1 group (*n* = 18) had the largest increase at T1 (4.8 ± 1.6), while the D-VAS ≥5 group (*n* = 8) had a smaller increase (2.1 ± 1.4), suggesting a ceiling effect. The lower baseline group showed more complete recovery at T2, with 83.3% (15/18) of procedures dropping below baseline compared to 37.5% (3/8) in the high baseline group (Figure 3). Tracheostomy patients (*n* = 9 procedures) had slightly lower D-VAS scores at T1 compared to intubated patients (5.8 ± 1.9 vs. 6.5 ± 2.1, *P* = 0.38), but the difference was not statistically significant.

Despite elevated SpO_2_ at T1 due to pre-oxygenation, D-VAS score change (T0 → T1) was positively correlated with HR change (*r* = 0.48, *P* < 0.001). Mild SpO_2_ decrease at T2 was correlated with persistent dyspnea (*r* = −0.35, *P* = 0.011).

### 3.5 Sensitivity analysis

After controlling for within-patient correlation using linear mixed-effects models, the fixed effect of time point remained highly significant (*F* = 118.93, *P* < 0.001). Random effects analysis showed that between-patient variance accounted for 23.4% of total variance (ICC = 0.234), indicating that individual differences influence dyspnea response.

## 4 Discussion

This prospective observational study found that closed-system suctioning was associated with transient but significant increases in self-reported dyspnea among awake mechanically ventilated patients. The mean D-VAS score increase of 3.6 (95% CI: 3.1–4.1) far exceeds the established MCID of 1–2 points (9), and 92.5% of patients experienced worsened dyspnea immediately after suctioning. These findings contribute to a growing body of evidence on symptom burden in the ICU and suggest that dyspnea monitoring during routine airway care procedures warrants further attention.

The neurobiological basis of this association may be understood through several hypothesized mechanisms. Neuro-mechanical uncoupling may be a key contributor: suctioning may cause alveolar collapse and reduced functional residual capacity (10), and although pre-oxygenation can temporarily maintain SpO_2_ at high levels (T1: 99.1 ± 1.3%), the acute decrease in lung volume could potentially lead to enhanced respiratory drive (11). If this mechanism is operative, patients may experience intense “air hunger,” potentially activating the insular cortex and limbic system (12, 13). Physical stimulation by the suction catheter, which shares neurophysiological overlap with the cough reflex (14, 15), may additionally contribute to sensations of “chest tightness.” It is important to note that these mechanistic interpretations are speculative in the context of the present observational data and cannot be directly confirmed without dedicated physiological monitoring such as esophageal manometry or diaphragmatic electromyography.

An important consideration in interpreting the SpO_2_ trajectory is the timing of pre-oxygenation. Because T1 was measured after FiO_2_ 1.0 had been delivered for 2 min, the elevated T1 SpO_2_ (99.1 ± 1.3%) primarily reflects the effect of pre-oxygenation rather than any oxygenation benefit conferred by suctioning itself. Crucially, this strengthens rather than weakens our central observation: despite maximal pharmacological oxygenation support being delivered concurrently with the procedure, patients reported substantial worsening of dyspnea. This pattern indicates that subjective respiratory distress during suctioning is dissociable from arterial oxygen saturation and cannot be prevented by oxygenation optimization alone.

The observed pattern in this study may suggest a dissociation between dyspnea and oxygenation level. Despite SpO_2_ reaching ≥98% in 83.0% of procedures at T1, patients still reported severe dyspnea (D-VAS: 6.4 ± 2.1). This pattern is broadly compatible with the “air hunger” theory proposed by Banzett et al. (16), which posits that dyspnea may arise primarily from mismatch between respiratory drive and lung volume, rather than oxygenation status *per se*. The acute increase in heart rate (mean +15.5 beats/min) may further reflect sympathetic activation potentially consistent with the affective dimension of dyspnea (17). However, the present study design does not permit causal conclusions about these physiological relationships.

Affective and anticipatory factors probably also shaped the D-VAS response, beyond neuro-mechanical and chemoreceptor-driven pathways. Anxiety is tightly linked to dyspnea in ventilated patients [odds ratio 8.84 in Schmidt et al. (4)], and the overlap between dyspnea and pain within emotion-processing networks involving the insula and anterior cingulate cortex described by von Leupoldt et al. (17) provides a plausible substrate for this interaction. In the setting of suctioning, visceral discomfort from catheter insertion, anticipation based on prior procedures, and a temporary loss of control could raise D-VAS scores even without an acute physiological respiratory derangement. This affective component may help explain slower recovery in patients with higher baseline dyspnea, for whom repeated unpleasant experiences could promote emotional sensitization. Future studies should consider concurrent anxiety assessment (e.g., Faces Anxiety Scale, FAS) alongside dyspnea measurement to disentangle the sensory and affective dimensions of the procedural response.

Qualitative literature shows that ICU survivors often describe the suctioning experience with terms such as “drowning” or “being strangled” (18), and airway-procedure-related discomfort has been identified as among the most distressing memories reported by ICU survivors (19), with associations with post-ICU post-traumatic stress disorder (20, 21). Although symptoms improved substantially during recovery (T2: 3.2 ± 1.7 vs. T1: 6.4 ± 2.1, *P* < 0.001), 15.1% of patients had not fully returned to baseline at 5 min (T2 vs. T0: *P* = 0.048), suggesting that some patients may require longer observation or additional intervention. Notably, mean SpO_2_ at T2 was slightly lower than baseline (96.8 vs. 97.2%, *P* = 0.031), but both values remain within normal oxygenation range, and the clinical significance of this change is limited.

The SpO_2_ and HR change patterns observed here are consistent with previous reports (22, 23), but the present study extends prior work by systematically quantifying changes in patients' subjective dyspnea experience alongside physiological parameters. Schmidt et al. (4) showed that dyspnea in mechanically ventilated patients was associated with increased anxiety and prolonged mechanical ventilation; the present findings are consistent with the notion that even routine nursing procedures may become acute triggers for dyspnea. Traditional objective observation tools such as the Respiratory Distress Observation Scale (24), while valuable, may not fully capture patients' subjective respiratory experience, highlighting the potential importance of direct patient-reported assessments (25). Subgroup analysis tentatively suggests that patients with lower baseline dyspnea had the largest acute increases but also recovered fastest (83.3% below baseline by T2), while those with higher baseline dyspnea showed smaller increases but slower recovery (37.5% fully recovered), a pattern that, if replicated in larger studies, could inform risk stratification.

Maggiore et al. (22) reported that endotracheal suctioning in ARDS patients caused substantial regional lung derecruitment and transient SpO_2_ changes, but their analysis focused exclusively on physiological endpoints without patient-reported symptoms. Our study extends this work by demonstrating that even when oxygenation is preserved or enhanced by pre-oxygenation, patients experience a clinically meaningful subjective dyspnea increase (mean D-VAS = 3.6, far exceeding the established MCID of 1–2 points). Similarly, Pedersen et al. (23) in their systematic review of suctioning practices emphasized physiological consequences and procedural safety but did not address patient-reported symptom burden—a gap our study directly addresses by quantifying self-reported dyspnea trajectories at three predefined time points. Compared to the purely observational dyspnea prevalence reported by Schmidt et al. (4) in ventilated patients at rest (median VAS = 5), the peak D-VAS of 6.4 immediately post-suctioning in our cohort suggests that procedural events may transiently push patients into more severe dyspnea states than their tonic baseline, underscoring the clinical importance of procedure-specific symptom monitoring.

### 4.1 Limitations

This study has multiple limitations that must be considered when interpreting the findings, which we present in the order of their potential impact on validity and generalizability.

First, methodological design constraints. This was a single-center convenience-sample study without an *a priori* sample size calculation. Although the large observed effect size for the primary outcome ($\eta_{p}^{2}=0.71$) suggests adequate *post hoc* statistical power for detecting the dyspnea response, *post hoc* power justification cannot substitute for prospective sample size estimation, and effect estimates may be inflated by selection of a single cooperative sample. Multicenter confirmatory studies with prospective power calculations remain essential before these findings can be considered definitive.

Second, repeated measures within patients. The inclusion of multiple procedures from the same patient introduces within-patient clustering and potential carry-over of emotional response across procedures. This was statistically addressed using linear mixed-effects models (ICC = 0.234, indicating that approximately 23.4% of total variance was attributable to between-patient differences), and the 24-h minimum inter-procedure interval was designed to limit anticipatory anxiety carry-over. Nonetheless, residual confounding from repeated exposure within the same patient cannot be entirely excluded. Future confirmatory studies should consider a strict one-procedure-per-patient design.

Third, assessor and operator non-independence. As described in the Methods, D-VAS assessment was performed by the bedside clinical nurse who also performed the suctioning procedure. Although standardized prompts, pointing-based scoring, and pre-study training were used to minimize reporter bias, complete blinding of the assessor from the procedure performer was not achieved. Patients may have moderated their responses in either direction in the presence of the procedure operator; the magnitude and direction of any residual reporter bias cannot be determined from the present data. Future studies should employ independent research observers blinded to procedural details.

Fourth, unmeasured physiological parameters. Tidal volume, respiratory rate, end-tidal CO_2_, esophageal pressure, diaphragmatic electrical activity, and ventilator-patient asynchrony events were not recorded. This represents the most important methodological gap, as it precludes direct quantification of neuro-mechanical uncoupling—the proposed mechanism most central to our interpretive discussion. The mechanistic interpretations presented in the Discussion should therefore be regarded as physiologically plausible hypotheses rather than direct empirical findings. Future studies should incorporate continuous ventilatory waveform analysis, esophageal manometry, or surface diaphragmatic EMG (26, 27).

Fifth, pharmacological confounders. Detailed records of sedative timing, dosing, and other psychoactive medications (analgesics, antidepressants, anxiolytics) prior to enrollment were not systematically cataloged. While the RASS 0 to +1 inclusion criterion ensured adequate awareness at the time of assessment, residual or fluctuating pharmacological effects on dyspnea perception cannot be fully excluded. The Glasgow Coma Scale (GCS) was not systematically collected; RASS served as the primary consciousness and sedation assessment tool, as it is specifically validated for ICU use and directly reflects the communicative capacity required for self-report.

Sixth, unmeasured chemoreceptor drivers. Arterial CO_2_ levels were not used as a pre-specified exclusion criterion or routinely measured at each time point. Severe hypercapnia could theoretically alter dyspnea perception through chemoreceptor-mediated drive enhancement, and the absence of this control represents an unmeasured confounder. However, the inclusion criterion of clinical stability (RASS 0 to +1, hemodynamic stability, absence of emergency indications) makes severe acute hypercapnia uncommon in the enrolled sample.

Seventh, ventilation mode and clinical heterogeneity. Enrolled procedures occurred under both A/C (47.6%) and PSV (45.2%) modes, which differ substantially in patient-ventilator interaction patterns and may differentially modulate the dyspnea response to airway perturbation. The study was not powered to detect mode-specific interactions. Similarly, post-operative patients were enrolled regardless of time since surgery, and surgical pain or anxiety unrelated to suctioning may have contributed to baseline dyspnea reports.

Eighth, assessment instrument limitations. The unidimensional D-VAS cannot distinguish between qualitatively distinct dyspnea sensations—“air hunger,” “work of breathing,” and “chest tightness”—which are known to have distinct neurophysiological substrates and emotional valences. The Multidimensional Dyspnea Profile would provide richer characterization but was not feasible in our acute-assessment context. Additionally, at T1 incompletely subsided cough reflex may have transiently amplified D-VAS scoring, and the 5-min T2 window may be insufficient to capture complete recovery in all patients; longer follow-up could reveal protracted distress in a subset.

Ninth, absence of long-term outcomes and single-center setting. Post-ICU psychological sequelae including PTSD, anxiety, and depression (28, 29) were not assessed, representing an important gap for future research linking acute procedural distress to long-term mental health outcomes. Furthermore, the study was conducted in a single tertiary ICU in Sichuan Province, China, with specific staffing models, patient demographics, sedation practices, and suctioning protocols. Generalizability to other settings requires external validation through multicenter studies. In particular, our institutional pre-oxygenation duration of 2 min exceeds the AARC-recommended 30–60 s, which likely produced more pronounced T1 SpO_2_ elevation than would occur under shorter pre-oxygenation regimens; the implications for the SpO_2_-dyspnea dissociation observed here are addressed in the Discussion.

Despite these limitations, the convergent direction of the primary findings (92.5% of procedures showing dyspnea worsening at T1, with effect size far exceeding MCID), the internal consistency across subgroups and physiological correlates, and the agreement with existing qualitative literature on ICU suctioning distress support the central observation of clinically significant procedural dyspnea independent of oxygenation status. The findings should be regarded as hypothesis-generating evidence motivating definitive multicenter investigation, rather than as definitive estimates of population-level effects.

### 4.2 Clinical implications

Integrating D-VAS assessment into routine suctioning care may be clinically valuable and deserves prospective evaluation. A practical model, aligned with existing palliative ICU care frameworks (30), would be to record dyspnea before suctioning, immediately afterward, and during recovery so that patients with disproportionate respiratory distress can be identified; given the observational and single-center nature of this study, this approach should be regarded as a hypothesis for future testing rather than a protocol-level recommendation.

Pre-oxygenation (FiO_2_ 1.0, 2 min) can temporarily maintain oxygenation, but the present data suggest it does not fully prevent dyspnea. A brief pre-procedure explanation remains a low-risk way to reduce anticipatory anxiety and is supported by existing guidelines (25, 31). Preventive analgesia is another candidate strategy for patients with higher baseline dyspnea or repeated suctioning needs, although respiratory depression risk must be weighed carefully (32). When dyspnea has not recovered by T2, lung recruitment maneuvers (33) and non-pharmacological interventions such as music therapy (34) provide plausible directions for study, even though RCT-level evidence specific to post-suctioning dyspnea is currently lacking. Clinically, the key point is that subjective dyspnea during suctioning is measurable, clinically significant, and partially dissociated from SpO_2_; patients' self-reported respiratory comfort therefore deserves consideration during routine airway care.

## 5 Conclusion

These observational findings suggest that closed-system suctioning is associated with significant, transient worsening of self-reported dyspnea in mechanically ventilated patients, with a pattern that appears independent of oxygenation status. The observed patterns are consistent with proposed mechanisms involving neuro-mechanical uncoupling and physical airway stimulation, though these interpretations require confirmation through studies incorporating dedicated physiological monitoring. In routine airway care, SpO_2_ should not be assumed to reflect respiratory comfort; future randomized controlled trials should test targeted strategies to reduce suctioning-related dyspnea while preserving procedural safety.

## Data Availability

The original contributions presented in the study are included in the article. Further inquiries can be directed to the corresponding author.
